# Supplementary material for: Unraveling the Antioxidant Activity of 2R,3R-dihydroquercetin
Source: Int J Mol Sci. 2023 Sep 18;24(18):14220. doi: 10.3390/ijms241814220 (PMC10532074; doi:10.3390/ijms241814220)
Supplement: Supplementary file 1 [file ijms-24-14220-s001.zip › Supplementary data S1.pdf]

Supplementary data S1

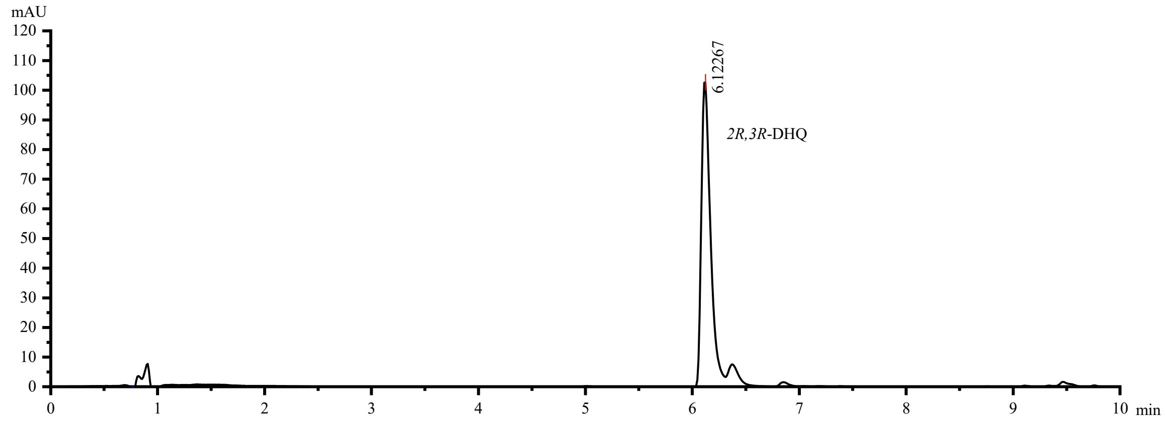

Figure S1. The LC-MS-ESI-IT-TOF/MS chromatogram of the *2R,3R*-DHQ standard (50 µM). The chromatogram presented were based on the detection at 290 nm.

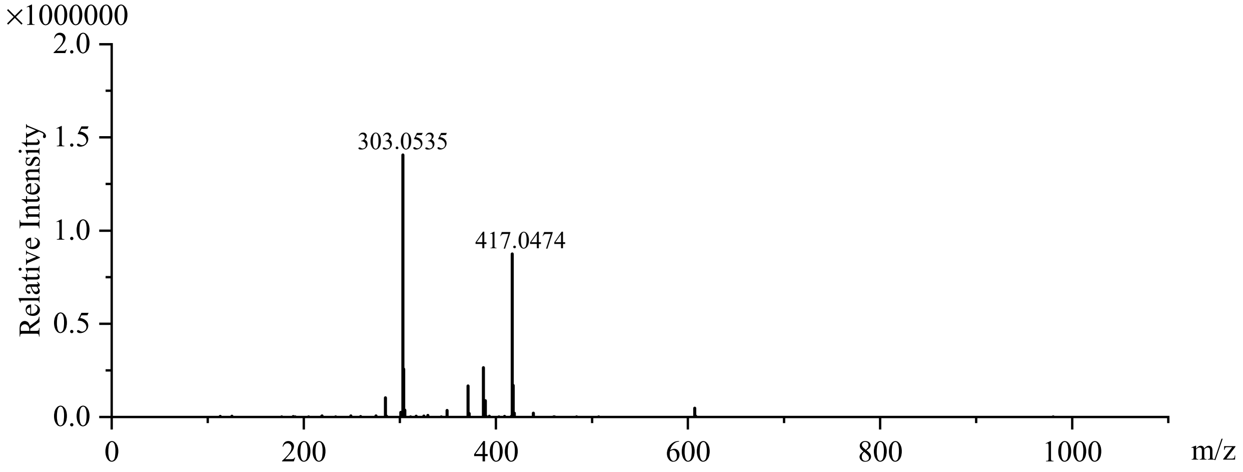

Figure S2. ESI-IT-TOF MS spectrum of the small peak observed in the chromatogram of the *2R,3R*-DHQ standard that appeared directly after the peak of *2R,3R*-DHQ (Figure 1). Based on this spectrum, this peak was attributed to *2R,3S*-DHQ.

In the LC-MS-ESI-IT-TOF/MS chromatogram of the *2R,3R*-DHQ standard (50 µM), there was a small peak alongside *2R,3R*-DHQ (Figure 1). The small peak had the same mass as *2R,3R*-DHQ, 303.05 m/z (Figure 2), and therefore the peak was attributed to *2R,3S*-DHQ that apparently present as a small impurity in the standard. That the *2R,3R*-

DHQ and *2R,3S*-DHQ have different retention time in our system is consistant with the results obtained by Li et al. [1] who by using a column comparable to the one as we used, also observed different retention times of the epimers. This corroborates that the *2R,3R*-DHQ can be detected separately from *2R,3S*-DHQ in our analytical system.

1. Li, Y., Su, H., Yin, Z.P., Yuan, E., Zhang Q.F. Metabolism, tissue distribution and excretion of taxifolin in rat. *Biomed. Pharmacother*. 2022, 150, 112959.
