# Supplementary material for: Unraveling the Antioxidant Activity of 2R,3R-dihydroquercetin
Source: Int J Mol Sci. 2023 Sep 18;24(18):14220. doi: 10.3390/ijms241814220 (PMC10532074; doi:10.3390/ijms241814220)
Supplement: Supplementary file 1 [file ijms-24-14220-s001.zip › Supplementary data S2.pdf]

Supplementary data S2

	2R,3R-Dihydroquercetin quinone	Open quinone	*2S,3R*-Dihydroquercetin quinone
Chemical structure	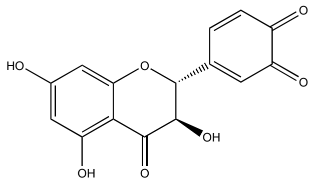	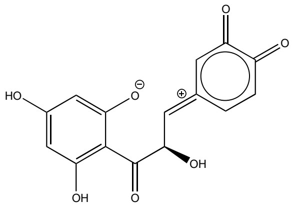	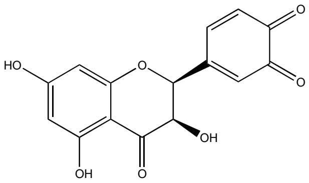
LUMO energy	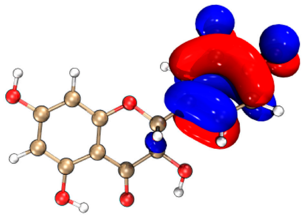 276.75 KJ/mol	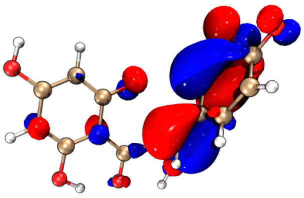 477.76 KJ/mol	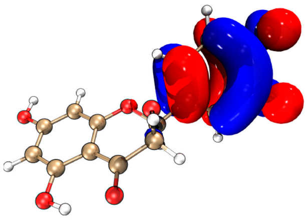 277.62 KJ/mol
HOMO energy	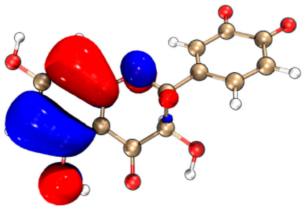 773.01 KJ/mol	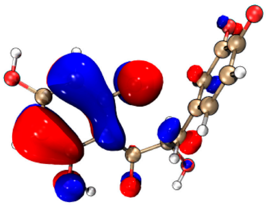 680.44 KJ/mol	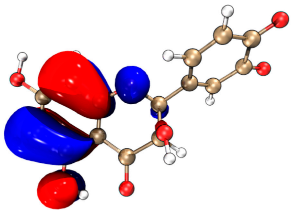 768.07 KJ/mol
Dual descriptor	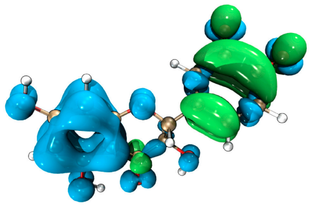	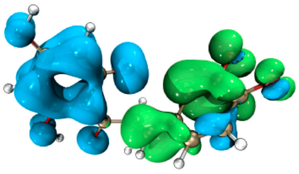	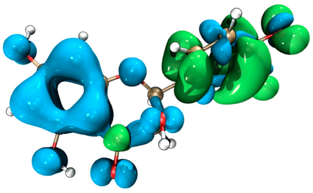

In the literature [2-4], the open quinone, instead of flat quinone intermediate, was proposed as intermediate in the epimerization of *2R,3R*-DHQ to *2S,3R*-DHQ. The structure of the open quinone that is difficult to draw in 2D, is shown in Figure 7. In Figure 7, it is shown how the energy of the LUMO orbital is mainly distributed in the three isomers of the DHQ quinone. For the open quinone, the energy of the LUMO is 477.76 KJ/mol and is primarily located in the B-ring. The LUMO of *2R,3R*-DHQ quinone and *2S,3R*-DHQ quinone are also mainly distributed in the B-ring, and their energies are 276.75 KJ/mol and 277.62 KJ/mol, respectively. Moreover, the HOMO-LUMO gap energy of *2R,3R*-DHQ quinone, open quinone and *2S,3R*-DHQ quinone are 496.26 KJ/mol, 202.68 KJ/mol and 490.45 KJ/mol, respectively. The calculations also showed that there is a relatively large difference in the single point energy between the open quinone and *2R,3R*-DHQ quinone or *2S,3R*-DHQ quinone, which is about 341.33 KJ/mol. The large difference in the LUMO energy, HOMO-LUMO gap energy and single point energy between open quinone and *2R,3R*-DHQ quinone or *2S,3R*-DHQ quinone combines to indicate that the transformation *2R,3R*-DHQ quinone or *2S,3R*-DHQ quinone with open quinone is not likely to happen. This corroborates that there was no substantial epimerization of *2R,3R*-DHQ in the experiment where *2R,3R*-DHQ was oxidized and subsequently the oxidation product was reduced by ascorbate.

References

2. Lundgren, L.N., Theander, O. Cis- and trans-dihydroquercetin glucosides from needles of Pinus sylvestris, *Phytochemistry*. 1988, 27 (3), 829-832.
3. Kiehlmann, E., Li, E.P. ChemInform abstract: Isomerization of dihydroquercetin, *ChemInform.* 1995, 58 (3), 450-455.
4. Osorio, E., Pérez, E.G., Areche, C., Ruiz, L.M., Cassels, B.K., Flórez, E., Tiznado, W. Why is quercetin a better antioxidant than taxifolin? Theoretical study of mechanisms involving activated forms., *J. Mol. Model*. 2013, 19 (5) , 2165-2172.
